# VirF Relieves the Transcriptional Attenuation of the Virulence Gene *icsA* of *Shigella flexneri* Affecting the *icsA* mRNA–RnaG Complex Formation

**DOI:** 10.3389/fmicb.2017.00650

**Published:** 2017-04-18

**Authors:** Mara Giangrossi, Anna M. Giuliodori, Chi N. Tran, Augusto Amici, Cristina Marchini, Maurizio Falconi

**Affiliations:** ^1^School of Bioscience and Veterinary Medicine, University of CamerinoCamerino, Italy; ^2^Food Science Department, Can Tho Technical – Economic CollegeCan Tho, Vietnam

**Keywords:** VirF, *icsA*, regulatory small RNAs, *Shigella* virulence, RNA–protein interaction, kissing complex

## Abstract

VirF is the master activator of virulence genes of *Shigella* and its expression is required for the invasion of the human intestinal mucosa by pathogenic bacteria. VirF was shown to directly activate the transcription of *virB* and *icsA*, which encode two essential proteins involved in the pathogenicity process, by binding their promoter regions. In this study, we demonstrate by band shift, enzymatic probing and cross-linking experiments that VirF, in addition to DNA, can also bind the *icsA* transcript and RnaG, an antisense non-coding small RNA that promotes the premature termination of *icsA* mRNA through a transcriptional attenuation mechanism. Furthermore, we show that VirF binds *in vitro* also other species of RNAs, although with lower specificity. The existence of VirF–RnaG and VirF-*icsA* mRNA complexes is confirmed in a pulldown assay carried out under experimental conditions that very close reproduce the *in vivo* conditions and that allows immobilized VirF to “fish” out RnaG and *icsA* mRNA from a total RNA extract. The VirF binding sites identified on both *icsA* mRNA and RnaG contain a 13 nucleotides stretch (5′**-**UUUUaGYcUuUau-3′) that is the RNA-converted consensus sequence previously proposed for the VirF–DNA interaction. Band-shift assays with a synthetic RNA molecule whose sequence perfectly matches the consensus indicate that this signature plays a key role also in the VirF–RNA interaction, in particular when exposed in a stem–loop structure. To further explore the *icsA*-RnaG-VirF regulatory system, we developed an *in vitro* test (RNA–RNA Pairing Assay) in which pairing between *icsA* mRNA and synthetic RNAs that reproduce the individual stem–loop motifs of RnaG, was analyzed in the presence of VirF. This assay shows that this protein can prevent the formation of the kissing complex, defined as the initial nucleation points for RNA heteroduplex formation, between RnaG and *icsA* mRNA. Consistently, VirF alleviates the RnaG-mediated repression of *icsA* transcription *in vitro*. Therefore VirF, by hindering the *icsA* transcript-RnaG interaction, exhibits an activity opposed to that usually displayed by proteins, which generally assist the RNA–RNA interaction; this quite uncommon and new function and the regulatory implications of VirF as a potential RNA-binding protein are discussed.

## Introduction

The gram-negative pathogen *Shigella flexneri* is the causative agent of human bacillary dysentery which causes about 1 million of deaths worldwide each year, the majority of which are children (Kotloff et al., 2013; Anderson et al., 2016). The VirF protein (30 kDa) of *Shigella*, encoded on the primary pathogenicity island carried by the large virulence plasmid, pINV, is the master activator leading to the invasivity phenotype (Di Martino et al., 2016a). Transcription of *virF* is thermoregulated and occurs only above the critical temperature of 32–34°C to prevent the expression of the virulence genes outside the host (Falconi et al., 1998). In fact, VirF, once synthesized, triggers a regulatory cascade, involving the second activator VirB, that, in turn, induces the expression of the virulence factors required for invasion and colonization of intestine epithelial cells by this pathogenic bacterium (Dorman and Porter, 1998; Prosseda et al., 2002; Parsot, 2005; Schroeder and Hilbi, 2008). Accordingly, mutants that do not express VirF are avirulent. For this reason, VirF is currently considered an ideal target for novel antibacterial agents for treating shigellosis (Koppolu et al., 2013; Emanuele and Garcia, 2015). While the regulation of *virF* gene expression has been extensively investigated (Falconi et al., 1998, 2001; Prosseda et al., 2004), to date, the protein has remained poorly characterized at biochemical level and little is known about the mechanism by which it activates transcription and its possible interactions with other regulators of virulence in *Shigella.*

VirF, as other transcriptional regulators of bacterial virulence (i.e., Rns/CfaD from *Escherichia coli*, ExsA in *Pseudomonas aeruginosa*, ToxT from *Vibrio cholerae* and LcrF in *Yersinia*), is a member of the AraC family (Egan, 2002) and consists of two domains: a N-terminal dimerization domain and a C-terminal DNA binding domain. Mutational analysis revealed that changes of key residues in the two helix-turn-helix (HTH) motifs predicted to interact with DNA, negatively impact the ability of VirF to activate the expression of target genes (Porter and Dorman, 2002). At present, the interaction with DNA and a direct role of VirF in stimulating promoter activity have been shown in the regulation of the virulence genes *virB* and *icsA* of *Shigella* (Tobe et al., 1993; Tran et al., 2011) and in the *yop* regulon of *Yersinia* (Wattiau and Cornelis, 1994). Recently, an opposed function was observed for a shorter form of VirF (21 kDa) that is able to bind its own promoter and to negatively autoregulate its expression (Di Martino et al., 2016b).

IcsA is a structural outer membrane protein which induces host actin polymerization at one pole of the cell, resulting in actin-tail formation that propels the bacterium from one cell to another (Bernardini et al., 1989; Agaisse, 2016). Recently, IcsA was also shown to promote the adhesion process to host cell, thus contributing further to the colonization of the intestinal mucosa (Brotcke Zumsteg et al., 2014). The *icsA* gene is subjected to a complex regulation that, in addition to VirF and the nucleoid protein H-NS, includes also a small non-coding RNA, named RnaG, encoded on the virulence plasmid pINV of *Shigella flexneri* (Giangrossi et al., 2010; Tran et al., 2011). RnaG (450 nt) is transcribed within the *icsA* gene and, acting as antisense, down-regulates *icsA* expression by a transcriptional attenuation mechanism. Briefly, RnaG is able to directly interact with *icsA* mRNA via a kissing complex establishment and to alter the structure of the nascent transcript, thus promoting the formation of a Rho-independent terminator that, in turn, leads to premature termination of *icsA* mRNA. Since the RnaG promoter is much stronger than the *icsA* promoter, the level of the antisense is always higher than that of the target mRNA, regardless the temperature and the VirF transcriptional stimulation (Giangrossi et al., 2010; Tran et al., 2011). Therefore, another mechanism is expected to act to relieve the antisense-mediated inhibition of *icsA* full transcription.

In this study, we identify a new role for VirF expanding and deepening our previous knowledge of the multifactor regulation of *icsA*. We demonstrate that VirF, in addition to DNA, is also able to bind RNA, in particular the small non-coding RnaG and *icsA* mRNA. In the specific cases of RnaG and *icsA* mRNA, the RNA–protein interactions appear so stable that these RNAs are selectively pulled down by VirF from a crude cellular RNA extract. By means of different techniques to study the RNA–protein interaction, we find that VirF exhibits several binding sites on both RnaG and *icsA* mRNA. Notably, VirF–RNA interaction is mediated by the preferential recognition of a 13-bp consensus sequence and binding sites are mostly located in the regions involved in formation of the kissing complex between RnaG and *icsA* mRNA. According to these results, we report that VirF bound at these sites can hamper the sense-antisense pairing and alleviate the RnaG-mediated repression of *icsA* transcription *in vitro*.

## Materials and Methods

### Bacterial Strains, General Procedures, and DNA Manipulations

The *hns* fragment of 530 bp (corresponding to the *hns* mRNA) was amplified by PCR using the oligonucleotides H238 and H239 and 10 ng of *E. coli* MRE600 chromosomal DNA as template. The DNA product was cloned downstream the T7RNA polymerase promoter into the EcoRI and BamHI sites of pSelectTM1 (Promega, Corp.). The resulting pSelect*hns* was used to synthesize the *hns* mRNA. The *virB* DNA fragment (212 bp) used to synthesize the leader region of *virB* mRNA was amplified by PCR from plasmid pBN1 (Adler et al., 1989) as template. The amplicon was obtained using the oligonucleotide T7VB (forward primer) that contains the T7 promoter followed by the sequence from position +1 to position +24 of *virB* and the oligonucleotide VB212 (reverse primer). VirF purification was performed as described by Tran et al. (2011). Radioactivity associated with DNA or RNA was detected and quantified by Molecular Imager (Bio-Rad, mod. FX) and oligonucleotides used in this study are listed in Supplementary Table S1.

### Electrophoretic Mobility Shift Assay (EMSA)

Electrophoretic mobility shift assay (EMSA) was made using the following RNAs: RnaG120 (Giangrossi et al., 2010), *icsA370*, mRNA (Giangrossi et al., 2010), *hns* (this study), *virB212* mRNA (this study), *cspD* mRNA (Giuliodori et al., 2004) and tRNA^Met^ (Sigma). Each RNA was transcribed with T7 RNA polymerase as indicated by Brandi et al. (1996) and labeled with T4 polynucleotide kinase (USB) and [γ-^32^P]-ATP as described by Giangrossi et al. (2010). M129 and M130 RNA oligonucleotides were labeled using T4 polynucleotide kinase and [γ-^32^P]-ATP. For M129/130 duplex formation, 5 pmol of M129 ^32^P-labeled were mixed with 15 pmol of M130 in Annealing buffer (50 mM KCl, 20 mM Tris HCl-pH 7.5). After incubating at 90–95°C for 1 min, the mixture (10 μl) was gradually cooled down to 49°C and kept at this temperature for 10 min before stopping on ice. The RNA duplex as formed was used in EMSA. Each 5′-end-labeled RNA (0.05 pmoles of mRNAs or 0.5 pmoles of oligonucleotides) was incubated for 10 min at 25°C in gel retardation buffer (20 mM Tris-HCl, pH 7.5, 50 mM KCl, 10% glycerol, 0.3 mg/ml Bovine Serum Albumin, 0.02% Nonidet P-40) with the indicated amounts of VirF and immediately loaded on a native 7% polyacrylamide gel electrophoresis (PAGE).

### Pulldown Assay

Pulldown assay was performed using the Dynabeads His-Tag Isolation & Pulldown (Novex). 50 μl of Dynabeads were mixed to 80 μg of purified histidine-tagged VirF in Buffer B (10 mM Tris HCl pH 7.5, 100 mM NaCl, 0.5 mM EDTA, 0.01% N-P40) and incubated on a roller for 30 min. A control sample containing only Dynabeads was processed in parallel. Then samples were washed two times with Buffer B and were incubated for 2 h at 4°C with 60 μg of total RNA extracted from *E. coli* HMG11 cells transformed with plasmid pGT1127 (Giangrossi et al., 2010). Subsequently, supernatants were collected and precipitated with ethanol whereas the beads were washed three times with Buffer B containing different NaCl concentrations (100, 200, and 400 mM). Finally, RNA bound to VirF was eluted from Dynabeads using Buffer B containing 300 mM imidazole, treated with phenol–clorophorm (1:1) and then ethanol-precipitated.

### RNA Footprinting Assay

Labeled RnaG or *icsA370* mRNA (∼1 pmol) denatured for 1 min at 90°C, were renatured for 5 min at 32°C and then incubated for 3 min without and with different concentrations of VirF in 15 μl of buffer A (20 mM Hepes-KOH, pH 7.5, 10 mM MgCl_2_, 50 mM KCl). RNA was treated with 0.075 U of RNase T1 (specific for unpaired G), 0.0025 U of RNase T2 (specific for unpaired C, A, and U) or 0.05 U of RNase V1 (specific for double-stranded RNA) for 3 min in presence of 1 μg of tRNA as competitor. The reaction was stopped with an equal volume of a phenol–chloroform mixture (1:1), and RNA was precipitated with ethanol. The reaction products were analyzed on 10% PAGE-urea gel in parallel with ΔT1 and OH- ladders (Donis-Keller et al., 1977).

### RNA–protein U.V. Cross-linking Assay

A mixture (8 μl) containing RnaG120 (0.5 pmol) and the [^32^P]-labeled G+1H oligonucleotide (2 pmol) was heated at 90°C for 1 min to denature RNA. Annealing of the primer with RnaG120 was carried out at 32°C for 5 min in buffer A (see above) in the presence or absence of VirF. The mixture was transferred to an ice-cold plate and U.V. irradiated for 1 min using the GS Gene-linker BioRad (180 mJ, 254 nm bulbs at ∼12 cm from the U.V. source). Finally the cross-linked RNA was primer-extended using the AMV Reverse Transcriptase as previously described (Giangrossi et al., 2010).

### RNA–RNA Pairing Assay (RRPA)

The *icsA* mRNA (2.5 pmol) and the [^32^P]-labeled RNA oligonucleotide GR4-48 (0.5 pmol) were denatured separately at 85°C for 1 min and placed on ice. The GR4-48 oligonucleotide was renaturated at the indicated temperatures for 5 min in the presence or absence of VirF before adding the denatured *icsA* mRNA. Pairing between *icsA* mRNA and GR4-48 was obtained prolonging the incubation for further 10 min at the same temperature in gel retardation buffer (see above) and then samples were loaded on 7% PAGE under native conditions.

### *In Vitro* Transcription Assay

The *in vitro* transcription assay was carried out by incorporating [α-^32^P]-UTP into mRNA using *the E. coli* RNA polymerase essentially as previously described (Giangrossi et al., 2010). NTPs concentrations were: ATP, GTP, and CTP 500 μM each, 20 μM of UTP and 0.2 μCi/μl of [α^32^P]-UTP. The *icsA* fragment (304 bp) to use as DNA template in the *in vitro* assay, was amplified by PCR with the oligonucleotides pair G-100/G+187 and plasmid pGT1129 (Giangrossi et al., 2010). After precipitation, the *icsA* transcription products (full-length and truncated transcripts), were analyzed on 7% PAGE-urea gel.

## Results

### VirF Binds RNA

**FIGURE 1:**
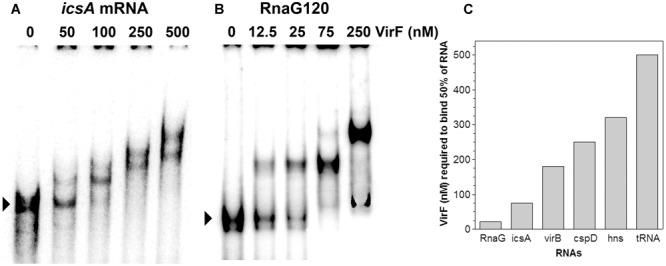
**Interaction of VirF with different RNAs.** The binding of VirF with *icsA370* mRNA **(A)** and RnaG120 **(B)** was analyzed by electrophoretic mobility shift assay (EMSA). The arrowheads indicate the electrophoretic mobilities of unbound RNAs. The interaction of VirF was also investigated using different RNAs (Supplementary Figure S1). The minimal concentrations of protein required to retard 50% of each RNA tested are reported **(C)**. EMSA experiments were carried out at least in duplicate.

VirF was shown, at least in few well-characterized genetic systems, to bind the promoter regions of its target genes and these interactions were required to affect their transcription (Tobe et al., 1993; Wattiau and Cornelis, 1994; Tran et al., 2011; Di Martino et al., 2016b). As explained before, we were looking for a regulatory mechanism able to relieve the RnaG-mediated inhibition of *icsA* full transcription. For this reason, we tested whether VirF, in addition to DNA, could also bind RNA. To verify this hypothesis, we investigated the possible interaction of VirF with *icsA* mRNA and RnaG by EMSA. These RNAs have been chosen because they belong to the *icsA–*RnaG genetic system which is regulated by VirF itself. As can be seen in **Figures 1A,B** VirF produces retarded bands with both the *icsA370* transcript, consisting of the whole leader region and part of the coding region, and RnaG120, carrying the entire antisense sequence (120 nt). About 50% of probe is recovered as RNA–protein complexes at 21 and 75 nM of VirF with RnaG120 and *icsA* mRNA, respectively. This result provides the first evidence that VirF is able to interact with RNA, displaying a ∼3.5-fold higher binding affinity for RnaG than that estimated for the *icsA* messenger RNA (**Figure 1C**). This finding led us to probe the possible interaction of VirF with a series of RNAs available in our laboratory, several of which unrelated to the *vir* system. The selected transcripts were: *hns* mRNA coding the nucleoid proteins H-NS, *virB* mRNA coding a transcriptional activator of virulence genes of *Shigella, cspD* mRNA coding a member of the cold shock protein family of *E. coli* and the *E. coli* tRNA^Met^. As shown in Supplementary Figure S1, VirF is able to interact with all the selected RNAs, although the nature of the RNA–protein complexes and the binding affinities are quite diverse and strongly dependent on the RNA species. In particular, half of *virB, cspD*, and *hns* transcripts is retarded at VirF concentrations ranging from 180 to 320 nM while tRNA^Met^ is shifted only at elevated protein concentrations (500 nM) (**Figure 1C**). These experiments demonstrate that, within the limit of accuracy of EMSA, VirF forms stable complexes with RnaG and *icsA* mRNA at a significant lower concentration than that required to bind the other control RNAs.

**FIGURE 2:**
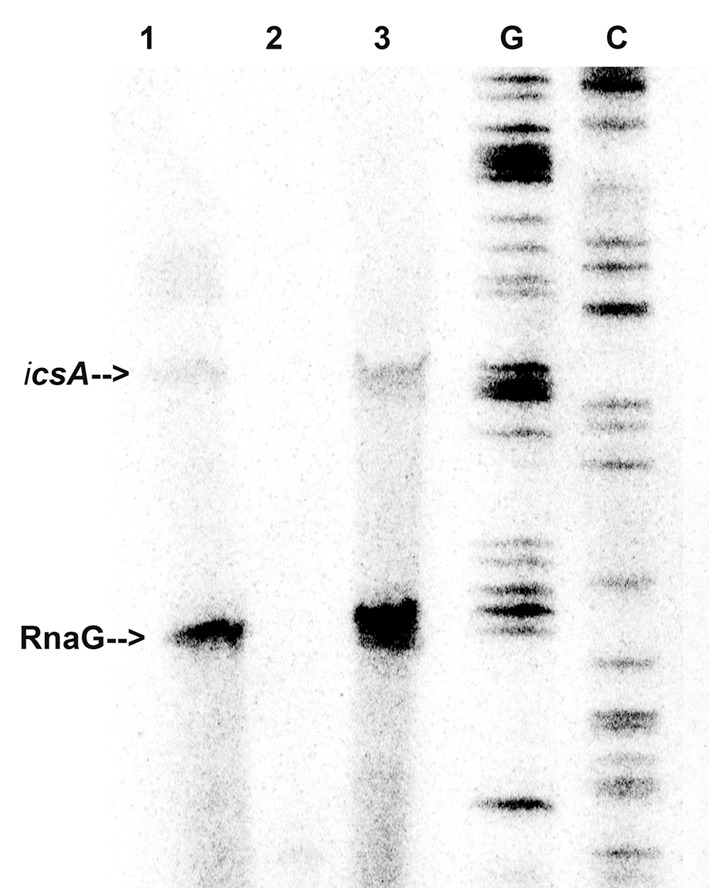
**VirF pulls down RnaG and *icsA* mRNA.** The pulldown experiment was carried out as described in Section “Materials and Methods” adding total RNA to both VirF immobilized on magnetic beads (lane 3) and beads alone as control sample (lane 2). After several washes, RNA was eluted from beads by removing the VirF–RNA complexes. Then, samples and 5 μg of total RNA extract (lane 1) were primer-extended using a mixture of primers G+110 and G+50 to detect *icsA* and RnaG, respectively (Giangrossi et al., 2010). Lanes G and C represent the sequencing reactions.

In light of this outcome, we tested whether immobilized VirF was able to “fish” out RnaG and *icsA* mRNA from a total RNA extract, to mimic the RNA–protein recognition occurring *in vivo.* Pulldown assay shows that both RnaG and *icsA* mRNA are recovered when bulk RNA is incubated with the VirF-coated magnetic beads (**Figure 2**, lane 3) while are undetected when empty beads (not complexed with the protein) are used as control (**Figure 2**, lane 2). The different levels of the two recovered RNAs reasonably reflect the higher expression of RnaG with respect to *icsA* mRNA, already observed in a previous work (Giangrossi et al., 2010). This differential expression, confirmed by the primer extension analysis of the total RNA not subjected to pulldown (**Figure 2**, lane 1), results from the transcriptional interference regulation controlling this genetic system (Giangrossi et al., 2010). Taken together, these data clearly indicate that, in addition to an intrinsic capability to stick to RNA, VirF specifically and selectively recognizes RnaG and *icsA* transcript, acting, at least with these RNAs, as a dedicated RNA binding protein.

### Identification of VirF Binding Sites on RnaG120 and *icsA370* mRNA

**FIGURE 3:**
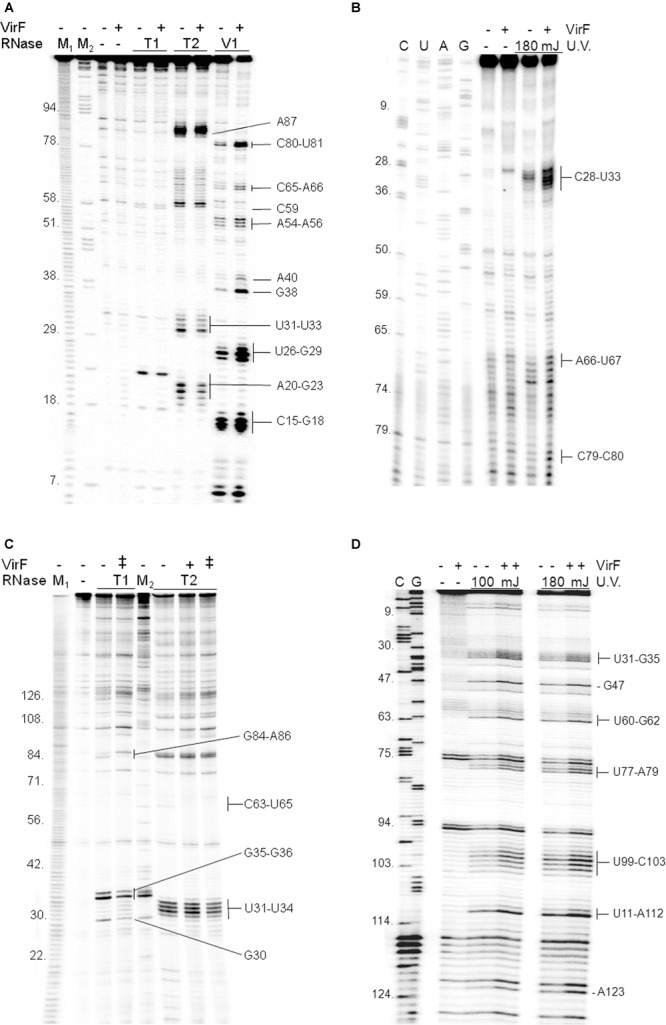
**Localization of VirF binding sites on RnaG120 and *icsA* mRNA.** RNA footprints were carried out, as described in Section “Materials and Methods,” on RnaG120 **(A)** and on the leader region (370 nt) of *icsA* mRNA **(C)**. RNases digestions were performed in the absence (–) or in the presence of 250 nM (+) of VirF in **(A)** and 250 nM (+) and 500 nM (++) of VirF in **(C)**. M_1_ and M_2_ represent OH- and ΔT1 ladders, respectively. U.V. cross-linking assays on RnaG120 **(B)** and *icsA* mRNA **(D)** were carried out without protein (–) and with 250 nM of VirF. Lanes C, U, A, and G correspond to sequencing reactions.

**FIGURE 4:**
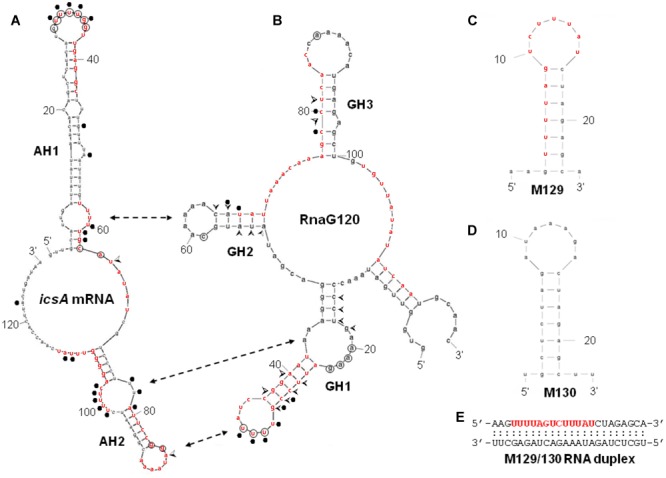
**Secondary structures of relevant RNAs.** VirF binding sites, as determined by RNA footprinting and U.V. cross-linking experiments shown in **Figure 3**, are reported on the secondary structures of RnaG120 **(A)** and leader region of *icsA370* mRNA **(B)**. GH1, GH2, GH3 motifs of RnaG120 and AH1 and AH2 domains of *icsA* mRNA have been identified by Giangrossi et al. (2010). Protected nucleotides from T1 and T2 RNases digestion by VirF are circled while positions of enhanced cleavage by V1 RNase are marked with arrowheads. The VirF–RNA U.V. cross-linked points are indicated with dots. The double-headed arrows indicates the stem–loop motifs involved in the formation of the kissing complex between RnaG120 and *icsA* mRNA (Tran et al., 2011). Secondary structures of M129 **(C)**, M130 **(D)**, and M129/130 RNA duplex **(E)**. The bases represent the sequences perfectly (M129) or partially (on *icsA* mRNA and RnaG) matching the RNA-converted consensus sequence determined by Wattiau and Cornelis (1994) for the interaction of VirF with DNA (5′- TTTaGYcTtTat-3′). The sequences reported in red on the RNA structures share at least 5/13 positions with the consensus. The regions 31–54 of *icsA* mRNA and 24–42 and 67–85 of RnaG contain two overlapping consensus-like sequences. Further details are given in Supplementary Figure S3.

Given that VirF tightly binds RnaG120 (**Figures 1, 2**), we performed RNA footprints using RNases T1, T2 and V1 to localize its potential binding sites on this target RNA. As seen in **Figure 3A**, VirF interaction causes several alterations of the sensitivity pattern of RnaG120 to RNases, most of which are localized on the GH1 domain (nucleotides 15–40). These changes include few positions protected against RNases T1 and T2 cleavages on single-stranded regions (loops and internal bulge) and many positions exhibiting an increased hydrolysis by RNase V1 on stem structures (**Figure 3A**). Overall, the GH2 stem–loop is generally less affected displaying only few nucleotides hyper-sensitive to RNase V1 cleavage at positions 54–56, 65, and 66. Even less pronounced effects are visible at GH3 hairpin where the susceptibility to RNases digestion of RnaG120 alone and that of the VirF–RNA complex does not significantly vary except for positions 80, 81, and 87 (**Figures 3A, 4B**). Downstream positions were not analyzed due to technical limitations. Localization of VirF binding sites on RnaG120 was confirmed also by means of RNA–protein U.V. cross-linking studies. In this assay, irradiation of RNA–protein complexes with ultraviolet light causes the formation of covalent bonds between VirF and RnaG120. These contact points can be mapped on RNA as stops (more intense bands) of reverse transcriptase in a primer extension reaction. The result of this experiment is shown in **Figures 3B, 4B**. As expected, VirF remains tightly cross-linked to GH1 (nucleotides 28–33) whereas very weak interactions are observed at domains GH2 (nucleotides 66 and 67) and GH3 (nucleotides 79 and 80).

In order to locate possible VirF binding sites on *icsA370* mRNA, footprinting and U.V. cross-linking experiments were carried out also on this transcript that, as RnaG, was efficiently retarded in gel mobility-shift assays (**Figure 1**) and pulled down (**Figure 2**) by VirF. As seen in **Figures 3C,D, 4A**, the two different techniques identify three regions as potential sites for the VirF–RNA interaction. These discrete sequences are: (i) the apical loop (nucleotides 30–36) of AH1 arm; (ii) the stem structure and the basal bulge (nucleotides 60–65) of AH1 arm; (iii) the basal bulge (nucleotides 77–79 and 99–103) and the apical loop (nucleotides 84–86) of AH2 arm. Therefore, the results of the U.V. cross-linking experiments, fully consistent with the enzymatic probing data, strongly suggest that the domains GH1 of RnaG and AH1 and AH2 of *icsA* mRNA are the preferential targets of VirF (**Figures 4A,B**).

### Sequence and Structure Specificity of VirF–RNA Interaction

**FIGURE 5:**
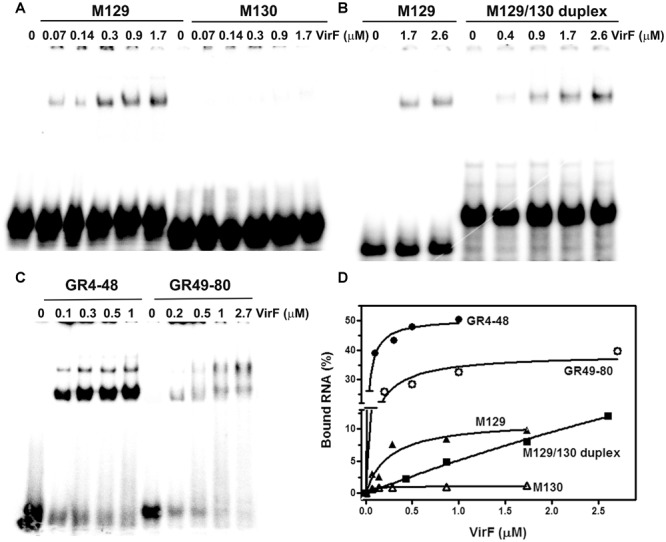
**VirF binds the GH1 domain of RnaG through the preferential recognition of a signature sequence.** EMSA was carried out, as a function of the indicated VirF concentrations, using the following ^32^P-labeled RNAs: M129 and M130 **(A)**; M129-130 duplex **(B)**; GR4-48 and GR49-80 **(C)**. Signals associated to bound RNAs were quantified and expressed as percentage of total radioactivity **(D)**.

By means of DNase I footprinting experiments on the *lc*rGVH-*yop*BD operon, Wattiau and Cornelis (1994) proposed, for the interaction of VirF with DNA, the 13-bp conserved sequence TTTaGYcTtTat (nucleotides with a frequency ≥ 60% are in uppercase and Y indicates pyrimidines). According to this study and to further investigate the determinants of the VirF–RNA interaction, we designed two synthetic RNAs, to be used in EMSA, that share the same stem–loop structure but carry (M129) or not (M130) the potential VirF recognition motif (**Figures 4C,D**). The gel shown in **Figure 5A** and the relative quantification (**Figure 5D**) clearly reveal that VirF binds M129, but not M130. However, a higher VirF concentration is required to shift the short M129 RNA compared to *icsA* mRNA and RnaG (**Figure 1**), indicating that the identified 13-bp consensus sequence is an element necessary but not fully sufficient for the interaction of VirF with the RNA. Notably, the sequences of M129 and M130 are complementary so that they could be annealed to yield a RNA duplex (**Figure 4E**). Comparing the binding capacity of VirF with the M129 and M129/130 RNAs emerges that this protein, particularly at lower concentrations (<1 μM), interacts less efficiently with its target sequence when this is buried within a RNA duplex (**Figures 5B,D** and Supplementary Figure S2). Overall, these data suggest that, in addition to the signature sequence, other factors (i.e., RNA structure, relative position of certain bases within the structure itself, number of close binding sequences) may contribute to the recognition process of specific RNA regions by VirF (see Discussion). Notably, AH1, AH2, and GH1 domains that were recognized as preferential binding sites of VirF, all contain consensus-like sequences (bases in red in **Figures 4A,B**) partially exposed in internal or apical loops.

### VirF Preferentially Targets the GH1 Domain of RnaG

We have previously shown that the ability of the non-coding RnaG (450 nt) to prematurely terminate *icsA* transcription resides in its antisense region (nucleotides 1–120) which hybridizes to the cognate messenger RNA. Complete pairing passes through the formation of an intermediate, the kissing complex, in which GH1 contacts AH2 whereas GH2 contacts AH1 whereas GH3 is dispensable (**Figure 4**). These interactions provide the initial nucleation points for RNA hetero-duplex propagation and are required for the establishment of *icsA* attenuation by RnaG (Giangrossi et al., 2010; Tran et al., 2011).

The evidence of a clear detectable VirF binding site on GH1 domain allowed to suppose that VirF, bound to RnaG, could interfere with the RNA–RNA interaction by hiding the sequences critical for the formation of kissing complex between sense and antisense RNAs. To verify this hypothesis and to study the interaction of VirF with the individual structural domains of RnaG, we designed two RNA oligonucleotides reproducing the GH1 (GR4-48) and GH2 (GR49-80) motifs. Initially, these synthetic RNAs were used to carry out EMSA experiments. As seen in **Figure 5C**, VirF retards both the GR4-48 and GR49-80 oligonucleotides indicating that its target sites are independent and binding occurs even when they are separated and individually assayed. Importantly, VirF exhibits, within the limit of accuracy of this type of analysis, a 2.5-fold higher capacity to interact with GR4-48 (∼50% bound RNA at 1 μM of protein) than with GR49-80 (∼45% bound RNA at 2.7 μM of protein) (**Figure 5D**). This result, in agreement with RNA footprints and U.V. cross-linking assays, confirms that GH1 represents the primary anchorage point recognized by the protein.

### VirF Hampers the Formation of the *icsA* mRNA–RnaG Duplex and Ultimately Alleviates the RnaG-Mediated Repression of *icsA* Transcription

**FIGURE 6:**
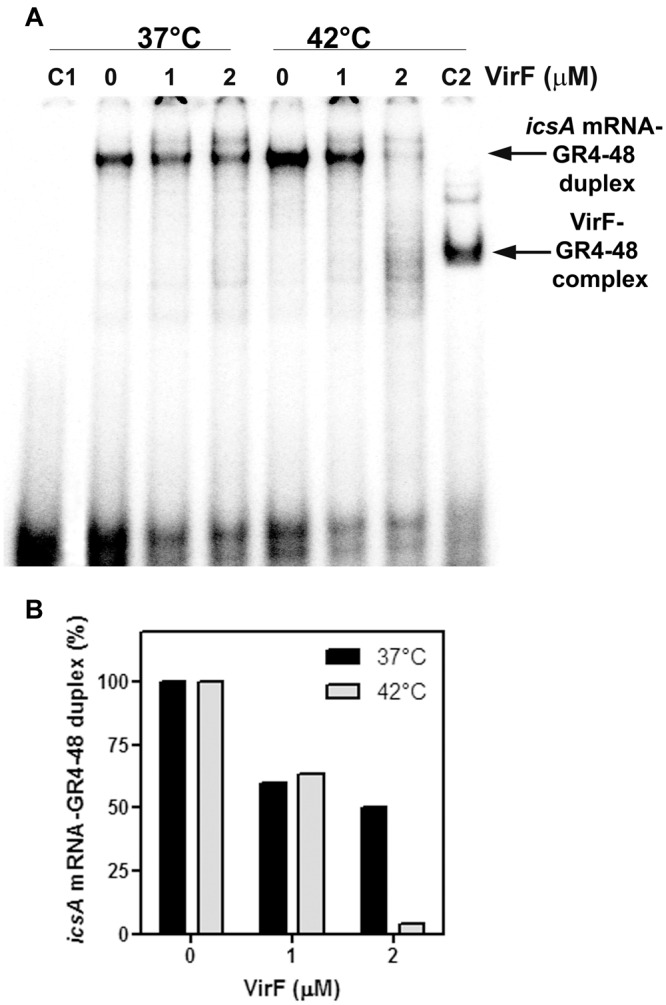
**VirF disfavors the interaction of the GH1 domain of RnaG with *icsA* mRNA. (A)** Pairing Assay was performed at 37 and 42°C as described in Section “Materials and Methods” using the indicated amounts of VirF and the GR4-48 RNA corresponding to the GH1 domain of RnaG. Lane C1 is a control sample containing the labeled GR4-48 oligo only. Lane C2 is a control sample containing the labeled GR4-48 RNA in presence of VirF (2 μM) but in absence of *icsA370* mRNA. **(B)** The radioactivity associated to the ^32^P-labeled GR4-48 paired with the “cold” *icsA370* mRNA has been quantified and expressed as percentage of the hetero-duplex formed without VirF (lanes marked with 0).

The outcomes so far presented prompted us to analyze the effect of the VirF–GH1 interaction on RnaG-*icsA* mRNA pairing. To this purpose, we developed an *in vitro* test called “RNA–RNA Pairing Assay” (RRPA), in which the heat-denatured *icsA370* messenger was added to a reaction mixture containing the 5′-end-labeled GR4-48 and VirF. Then, the free GR4-48 oligonucleotide and that hybridized with the *icsA* mRNA were electrophoretically resolved under native conditions and radioactivity associated with the RNA hetero-duplex was quantified. RRPA was performed at two temperatures (37 and 42°C) and VirF concentrations (**Figure 6A**). Increasing amounts (from 1 to 2 μM) of VirF cause a progressive reduction of bound GR4-48 suggesting that this protein blocks the interaction between the GH1 domain of RnaG and *icsA* mRNA. This effect is clearly detectable at 37°C and becomes very pronounced at 42°C (**Figure 6B**).

**FIGURE 7:**
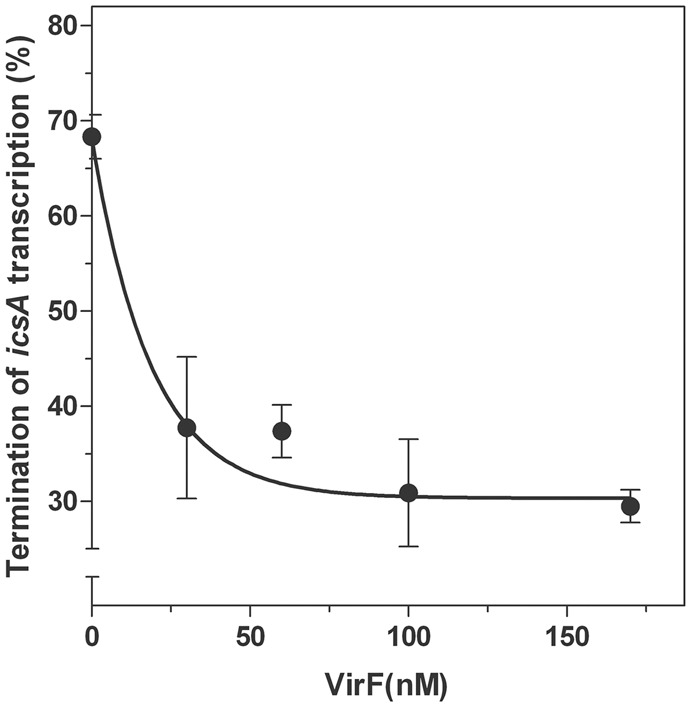
**Effects of VirF on the RnaG-mediated repression of *icsA*.** The *in vitro* transcription test programmed with *icsA* was performed, as described in Section “Materials and Methods,” in presence of 2 pmol of RnaG120 as a function of increasing amounts of VirF. Full-length and truncated mRNAs transcribed in the presence and in the absence of VirF were resolved in 7% PAGE-urea gel. The termination products are expressed as percentage of the total transcription. Data represent the average of at least three independent experiments and standard deviation is indicated.

Next, by means of an *in vitro* assay, we investigated the transcriptional attenuation of *icsA* by RnaG120 as a function of increasing concentrations of VirF. According to Giangrossi et al. (2010), RnaG determines the appearance of a truncated *icsA* transcript due to the formation of an intrinsic terminator on the target mRNA. Under the experimental conditions used (**Figure 7**), termination products promoted by RnaG120 represented about 70% of all transcription events. VirF addition causes lower levels of transcriptional termination of *icsA* (30%), indicating that this protein is able to alleviate, albeit not completely, the repressive action of RnaG possibly by sequestering the RnaG itself and its target mRNA.

## Discussion

In the last decade, there has been an explosion in the discovery of bacterial small regulatory RNAs (sRNAs) and in the identification of their targets. Most of characterized sRNAs function by base-pairing with their target mRNAs and fall into two categories: *trans*-encoded and *cis*-encoded RNAs. *trans*-encoded sRNAs have multiple target mRNAs and regulation is exerted via the interactions of short and imperfect complementary sequences. *cis*-encoded sRNAs, also known as antisense RNAs (asRNA), are transcribed from the opposite DNA strand of established coding sequences and hence share fully complementary with their targets. In the majority of cases, both *trans*- and *cis*-encoded small RNAs act at post-transcriptional level by inhibiting the function of target RNAs. Multiple aspects concerning small regulatory RNAs in bacteria are reviewed in Georg and Hess (2011), Gottesman and Storz (2011), Storz et al. (2011), Caldelari et al. (2013), Brantl and Brückner (2014), Bobrovskyy et al. (2015), Papenfort and Vanderpool (2015), Wagner and Romby (2015). Despite the large number of sRNAs recently discovered by deep-sequencing based methods Sharma and Vogel (2014), clear physiological roles and mechanisms of action have been established only for a small number of them. Even less is known about proteins associated with regulatory sRNAs and how these proteins may modulate their activities.

Few years ago, we have identified the first regulatory antisense RNA, encoded by the virulence plasmid pINV of *S. flexneri.* This RNA, named RnaG, represses by a transcriptional attenuation mechanism, the expression of IcsA (Giangrossi et al., 2010), an invasion protein required for host colonization by pathogenic bacteria. Currently, three other small RNAs, that regulate virulence genes, have been identified and characterized in *Shigella* (Fris and Murphy, 2016). In addition to RnaG, we found that the activity of *icsA* promoter is modulated by three other mechanisms (Giangrossi et al., 2010; Tran et al., 2011): (i) direct stimulation of *icsA* transcription by VirF at 37°C; (ii) H-NS-mediated inhibition of *icsA* mRNA synthesis; (iii) transcriptional interference, being *icsA* and RnaG promoters convergent and 120 nt apart.

A deep investigation on the *icsA*–RnaG genetic system with a focus on the role of VirF, has led us to identify a new property for this protein. In fact, VirF known so far to solely bind DNA, can also interact with RNA as demonstrated in this work. Due to the complexity of the overall regulatory mechanism governing *icsA* expression and to the objective difficulties to evaluate the individual and combined contributions of each factor *in vivo*, we chose to study this new feature of VirF by means of an *in vitro* approach. Gel mobility shift assays show that VirF binds *icsA* mRNA and RnaG very tightly although it is able to associate also with other RNAs (*hns, virB, cspD mRNAs* and tRNA) (**Figure 1** and Supplementary Figure S1). Within the limit of accuracy of this technique, binding capacity varies in a very broad range from ∼20 nM for RnaG to ∼500 nM for tRNA, suggesting that the nature (i.e., sequence and structure) of the RNA itself mediates the interaction of VirF. Searching for the determinants of the VirF–RNA interaction, we performed an *in silico* analysis that revealed the presence of several sequences on both RnaG and *icsA* mRNA, shearing a similarity with the degenerated logo TTTaGYcTtTat, previously identified by Wattiau and Cornelis (1994). Notably, the positions of these consensus-like sequences almost completely match the VirF binding sites mapped on these two RNAs (**Figure 4**). Furthermore, their primary role is validated by the fact that VirF binding capacity is strongly impaired with a RNA oligonucleotide (M130) that lacks this 13-bp conserved motif. Accordingly, VirF shows a higher binding affinity (∼2.5-fold) for domain GH1 of RnaG, which contains two overlapping sequences matching in 6/13 and 9/13 positions the consensus, than for domain GH2, that has only a highly degenerated motif (5/13 matching positions), as clearly proved by the EMSA experiment carried out with the separated domains (**Figure 5**). Finally, similar sequences, although with lower frequency and conservation, have been found also the other RNAs retarded by VirF (Supplementary Figures S1, S3). A deeper inspection of prominent VirF sites on RnaG120 and *icsA* mRNA evidences that they are characterized by a U-rich single-stranded region (loops or bulges) adjacent to a helix which places part of the recognized bases into a structural context that reproduces the natural condition occurring in DNA. This circumstance is particularly evident at the apical hairpins of GH1 and AH1 and at the internal bulge of AH2 (**Figure 4**). In this context, the unpaired U-stretch seems to play a key role. In fact, VirF binds better the target sequence arranged in a stem–loop structure with respect to the same sequence entirely paired in a RNA duplex (**Figure 5**). Thus, multiple elements (structurally distinct motif of RNA, exposition of given bases and adjacent signature sequences) can concomitantly contribute to the recognition process of RNA by VirF.

**FIGURE 8:**
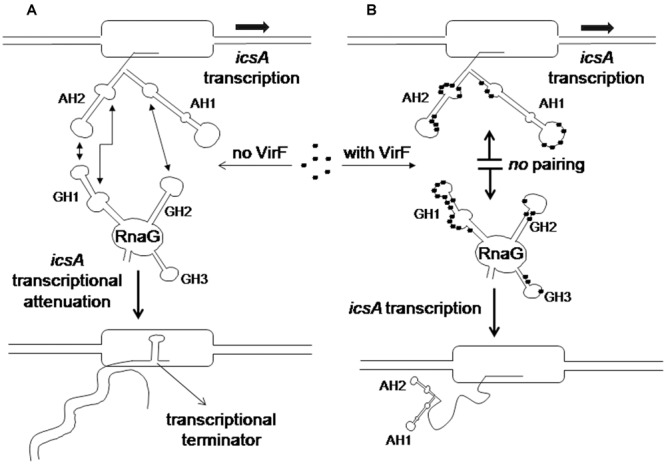
**Regulatory mechanism of the *icsA-*RnaG-VirF system. (A)** In the absence of VirF, RnaG forms a hetero-duplex with the nascent *icsA* message. This results in the formation of a terminator hairpin, transcription is attenuated and an abortive RNA is released. **(B)** When VirF is present, it interacts with both *icsA* mRNA and RnaG preventing the sense–antisense pairing. Thus the 5′ region of the newly transcribed *icsA* transcript folds into the AH1 and AH2 stem–loop motifs that, acting as an antiterminator structure, allows transcription of the *icsA* full-length mRNA.

RNA loop–loop interactions are frequently used to trigger the initial recognition between two RNA molecules. Such pairing, involving exposed nucleotides, originates the kissing complex (Wagner and Romby, 2015; Durand et al., 2016). In a previous study, we have elucidated the secondary structures of the 5′-end of *icsA* mRNA (∼150 nt) and RnaG120, identifying those nucleotides required for establishing the kissing complex between the sense and antisense RNAs. In particular, a mutational analysis of RnaG revealed that: (i) the apical loop of GH2 pairs with the basal bulge of AH1; (ii) the apical loop and the internal bulge of GH1 anneal with structurally similar motifs present on AH2; (iii) the GH3 domain is not involved in *icsA* mRNA–RnaG pairing. The secondary structures of these cognates RNAs, including their initial contact points as determined by Tran et al. (2011), are shown in **Figure 4**. As mentioned above, our analysis of the VirF–RNA interaction, overall suggests that this protein has two primary target sites: the GH1 domain of RnaG and (being RnaG the natural *icsA* antisense) its complementary region on *icsA* mRNA, the AH2 arm. These regions actively participate to the formation of kissing complex. The schematic model of **Figure 8** shows that, by masking at least one of the pairing sequences (GH1 and AH2), independently of its location on RnaG or *icsA* mRNA, VirF could prevent the formation of a functional kissing complex thereby allowing the synthesis of the full-length *icsA* transcript. Furthermore, several points of hypersensitivity to V1 RNase cleavage (a double-stranded specific ribonuclease) induced by VirF interaction are found at GH1 and GH2 stems of RnaG (**Figure 3A**). This observation suggests that VirF may contribute to stabilize the RNA duplex thus trapping RnaG into its native structure, a rigid state functionally unable to hybridize with *icsA* mRNA. Our conclusions are supported by three main lines of evidence. First of all, RnaG and *icsA* mRNA are selectively pulled down by VirF from a total RNA preparation under competitive conditions that close mimics those existing *in vivo* (**Figure 2**). Secondly, VirF is able to alleviate the termination of *icsA* transcription *in vitro* by targeting RnaG (**Figure 7**). Finally, the yield of the hetero-duplex formed between the GH1 domain of RnaG and *icsA* mRNA increases as a function of temperature from 32 to 42°C, as shown in the RRPA assay of Supplementary Figure S4. This means that at temperatures ≥ 37°C there is more *icsA* mRNA–RnaG complex, a condition that represses *icsA* transcription. However, VirF is able to hamper this sense-antisense pairing in a temperature-mediated manner, being its effect more pronounced at temperatures ≥ 37°C (RRPA assays shown in **Figure 6** and Supplementary Figure S4). The relieve of the RnaG-mediated attenuation of *icsA* caused by VirF binding may ensure that bacterial invasion is maintained *in vivo* when the host responds to severe *Shigella* infection with high fever. In summary, transcription of *icsA* can be activated at host temperature by VirF through two not mutually exclusive mechanisms: by directly stimulating the activity of *icsA* promoter (Tran et al., 2011) and by hindering the formation of the repressive *icsA* mRNA–RnaG complex (this study).

Except for VirF, the *icsA*–RnaG system is properly regulated in absence of other RNA helper proteins and is likely Hfq-independent (Giangrossi et al., 2010). In addition to this genetic system, two other cases of dedicated RNA binding proteins, modulating antisense RNAs activity have been reported. These proteins are Rom (Rop) and FinO involved in the replication control of ColE1 and F plasmids, respectively. Whereas Rom stabilizes the kissing product formed by RNA I and RNA II through conversion of an unstable early intermediate to a more stable complex (Eguchi and Tomizawa, 1990), FinO facilitates the loop-to loop-interactions between the asRNA FinP and *traJ* mRNA (Arthur et al., 2003). Unlike these proteins that assist the RNA–RNA interaction, VirF negatively affects the fate of the kissing complex formed by RnaG and *icsA* transcript. This is quite an uncommon function for a protein able to bind RNA. To our knowledge, the *icsA*-RnaG-VirF three components system is the first well-studied case in which this kind of interplay takes place, providing new mechanistic aspects for the overall comprehension of riboregulation in bacteria.

Unlike many other DNA binding proteins, VirF seems capable of recognizing either the DNA sequences and their RNA counterparts. Although this property is rather uncommon, it is worth mentioning that also two important DNA binding proteins, namely H-NS (Brescia et al., 2004; Park et al., 2010) and HU (Balandina et al., 2001), were found to be able to bind both types of nucleic acid. Our experiments seem to indicate that VirF recognizes the same consensus binding sequence on both DNA and RNA. To collect some other information on this issue, we performed an *in silico* analysis using program RNAbindRplus (Walia et al., 2014), that is able to predict RNA-binding residues from primary protein sequences by combining Support Vector Machine and Homology-based methods. The result of this analysis indicates that R192, H193, H212, S236, P237, Y239, N245, T251, P252, K253, and K254 are the residues possibly involved in the binding to RNA. Notably, these residues are located in the two H-T-H motifs of this AraC family member and two of these residues, namely H193 and Y239, were found to be critical for the binding of VirF to DNA (Rhee et al., 1998; Porter and Dorman, 2002).

Microarray experiments using *E. coli* and *Shigella* cells expressing or not VirF have led to the identification of a large set of genes common to both bacteria, that are activated either directly or indirectly by this regulator (Barbagallo et al., 2011). These observations, in connection with our data showing the interaction of VirF with different RNA species (i.e., mRNAs, small RNA, tRNA), strengthen the emerging idea that this protein might be a powerful and flexible global regulator acting at both transcriptional and post-transcriptional levels and participating to diverse regulatory networks.

## Author Contributions

MG, CT, AG, and MF designed and performed most of the experiments giving an important contribution also to the analysis and interpretation of data. AA and CM carried out some experiments. MF, AG, and MG have been dealing with the preparation of figures, drafting the work and revising it critically.

## Conflict of Interest Statement

The authors declare that the research was conducted in the absence of any commercial or financial relationships that could be construed as a potential conflict of interest.
