# Supplementary material for: VirF Relieves the Transcriptional Attenuation of the Virulence Gene icsA of Shigella flexneri Affecting the icsA mRNA–RnaG Complex Formation
Source: Front Microbiol. 2017 Apr 18;8:650. doi: 10.3389/fmicb.2017.00650 (PMC5394118; doi:10.3389/fmicb.2017.00650)
Supplement: Supplementary file 1 [file Presentation_1.pdf]

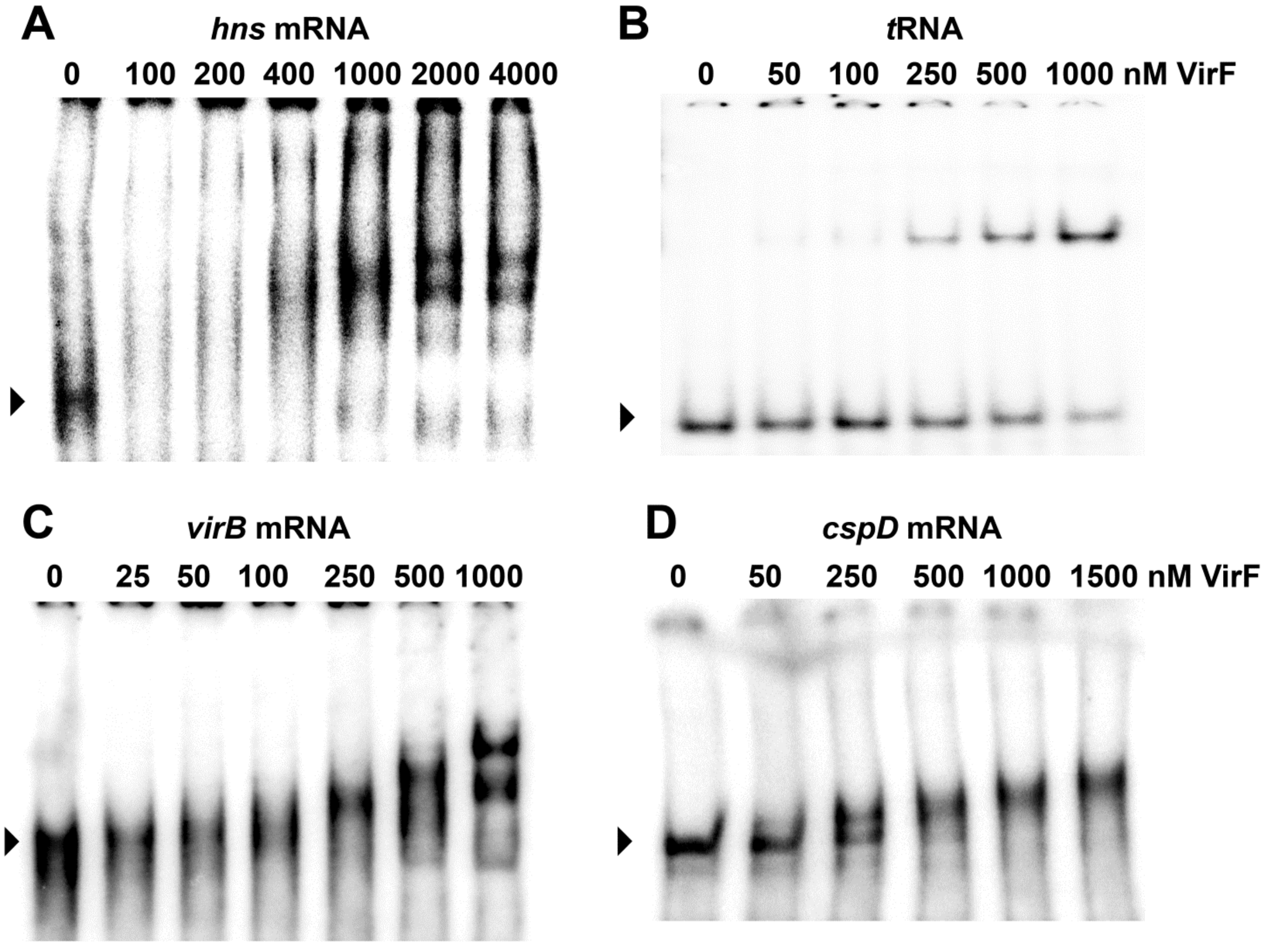

Figure S1. VirF binds various RNAs. EMSA experiments were carried out with the following RNAs: *hns* mRNA (**A**), tRNA^{Met} (**B**), *virB212* corresponding to the 5’-end of mRNA (**C**) and *cspD* mRNA (**D**). The concentrations of protein required to retard 50% of each RNA tested are reported in Figure 1C.

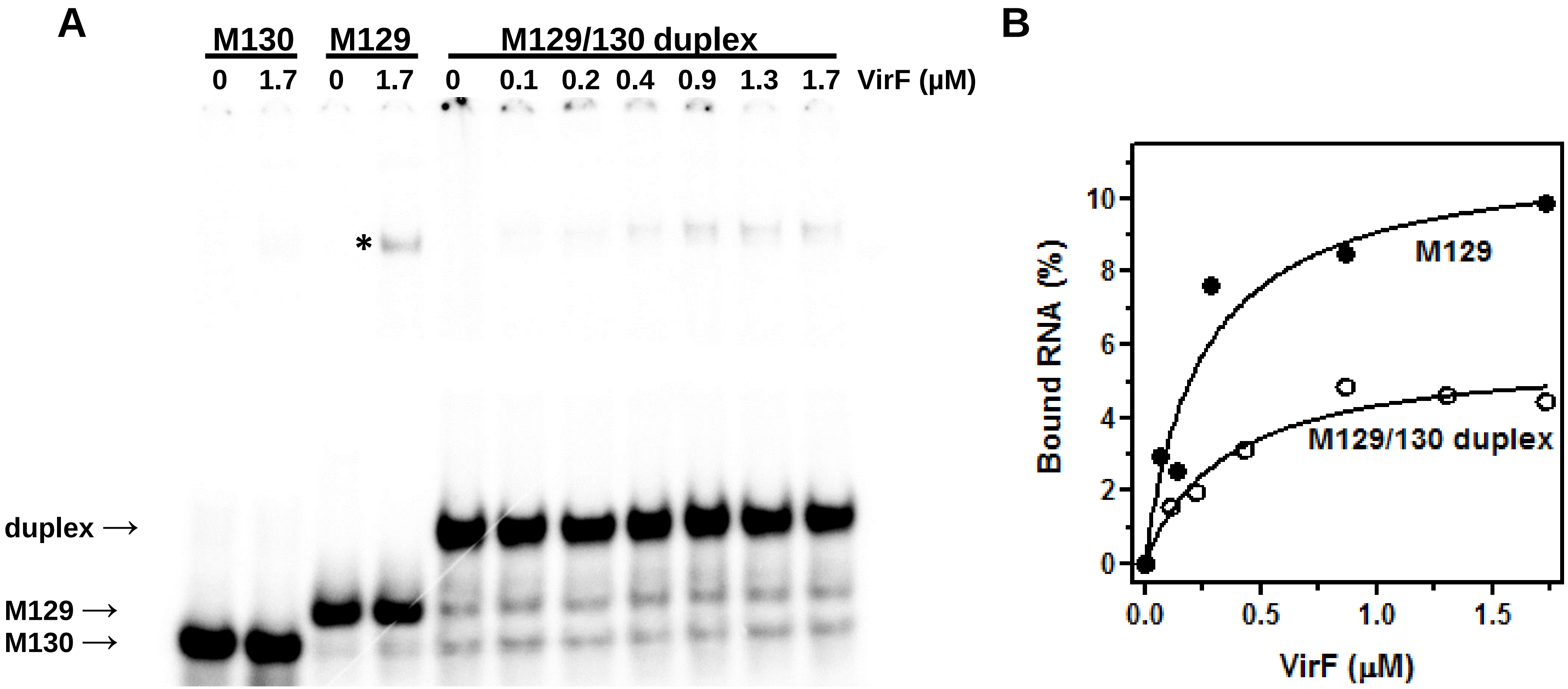

Figure S2. Binding of VirF to its target sequence. (**A**) EMSA was carried out essentially as described in the body text and in the legend of Figure 5 using the following ^{32}P-labelled RNAs: M129, M130 and M129/130 duplex. (**B**) Signals associated to bound M129/130 RNA duplex were quantified and expressed as percentage of total radioactivity whereas the M129 curve is plotted using the values of Figure 5D. The reproducibility between these experiments is validated by the fact that 1.7 μM of VirF retards 9 % of M129 (band indicated with the asterisk).

A)

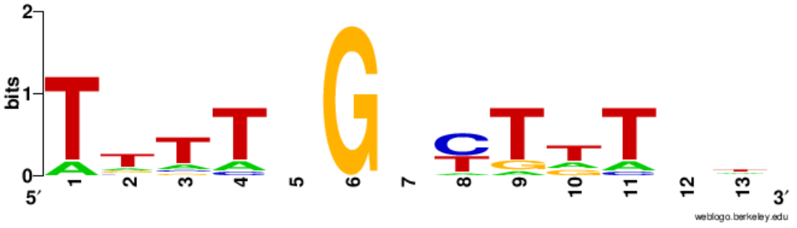

B)

***icsA* mRNA (*S. flexneri*)**

5'GUUGCAUUGAUAUAUAACACAGCUCUCAUGUUUUGGUUGAGGCUUUGUUUAAUAUGUUUUUGCAUA
UAUCGUCCCUUUAUUCCGGAUAAAACGGAAUCUUUUCAGGGGUUUAUCAACCACUUACUGAUAAUAU
AGUGCAUGAAUCAAAUUCACAAAUUUUUUUGUAAUAUGACCCAAUGUUCACAGGGGGGGGCCGGAGA
AUUACCUACGGUAAAGGAAAAAACAUGCAAAUUGUCUUUUUCUCCUUUUGUUGUUGGUGCAUCCCUG
UUGCUCGGGGGGCCAAUAGCUUUUGCUACUCCUCUUUCGGGUACUCAAGAACUUCAUUUUUCAGAGG
ACAAUUAUGAAAAAUUAUUAACACCUGUUGAUGGACUUUCUCCCUUGGGAGCUGGUGAAGAUGGAAU.
..3'

***RnaG* (*S. flexneri*)**

5'GUGGUUGAUAAACCCCUGAAAAGAUUCCGUUUUAUCCGGAAUAAAGGGACGAUAUAUGCAAAAACA
UAUUAAACAAAGCCUCAACCAAAACAUGAGAGCUGUGUUAUAUAUCAAUGCAAC...3'

***virB* mRNA (*S. flexneri*)**

5'ACACUGCAUUUAACUUUUGUCAAUAUAAAUCAAUAUGAAUAAACAGGGUGUGAUAUGGUGGAUUUG
UGCAACGACUUGUUAAGUAUAAAGGAAGGCCAAAAGAAAGAGUUUACACUCCAUUCUGGUAAUAAAG
UUUCCUUUAUCAAAGCCAAGAUUCCUCAUAAAAGGAUCCAAGAUUUAACCUUCGUCAACCAAAAAACG
AAUGUACGCGA...3'

***cspD* mRNA (*E. coli*)**

5'AGAGUUGGCGAGGUUUUGAACAGCCCCCUCUCUGACCCCGGUUUAUUCCAUCUUACUUGUAUAAGA
UUUGCGAAGGAUGUCGAAGCAUGGAAAAGGGUACUGUUAAGUGGUUCAACAAUGCCAAAGGGUUUGG
UUUCAUCUGCCCUGAAGGCGGCGGCGAAGAUAUUUUCGCUCAUUAUUCCACCAUUCAGAUGGAUGGU
UACAGAACGCUAAAAGCUGGACAAUCCGUUCAGUUUGAUGUCCACCAGGGGCCAAAAGGCAAUCACGC
CAGUGUUAUUGUGCCCGUCGAAGUAGAAGCGGCAGUCGCAUAG 3'

***hns* mRNA (*E. coli*)**

5'AACAAACCACCCCAAUAUAAGUUUGAGAUUACUACAAUGAGCGAAGCACUUAAAAUUCUGAACAAC
AUCCGUACUCUUCGUGCGCAGGCAAGAGAAUGUACACUUGAAACGCUGGAAGAAAUGCUGGAAAAAU
UAGAAGUUGUUGUUAACGAACGUCGCGAAGAAGAAAGCGCGGCUGCUGCUGAAGUUGAAGAGCGCAC
UCGUAAACUGCAGCAAUAUCGCGAAAUGCUGAUCGCUGACGGUAUUGACCCGAACGAACUGCUGAAU
AGCCUUGCUGCCGUUAAAUCUGGCACCAAAGCUAAACGUGCUCAGCGUCCGGCAAAAUAUAGCUACGU
UGACGAAAACGGCGAAACUAAAACCUGGACUGGCCAAGGCCGUACUCCAGCUGUAAUCAAAAAAGCAA
UGGAUGAGCAAGGUAAAUCCCUCGACGAUUUCCUGAUCAAGCAAUAA 3'

***tRNA$_{fmet}$* (*E. coli*)**

5'CGCQGGQUGGAGCAGCCUGGDAGCUCGUCGGGCUCAUAACCCGAAGGUCGCCGGUPHICAAAUCCGG
CCCCCGCAACC 3'

Figure S3. Localization of VirF binding sites in the tested RNAs. (A) Logo representation of the VirF binding motif derived from the multiple alignment published by Wattiau and Cornelis (1994). The logo was drawn using WebLogo (http://weblogo.berkeley.edu/logo.cgi). **(B)** Sequences of the RNAs tested in this work for VirF bindig. The stretches highlited in light gray and dark gray represent the *in silico* predicted VirF binding sites matching at least 4/9 and 6/9 of the consensus sequence, respectively. The bases matching those occurring most frequently and less frequently in theVirF logo are indicated in blue and black, respectively. The analysis was performed allowing one mismatch out of 13 bases in each stretch. When present, the initiation codon is indicated in magenta.

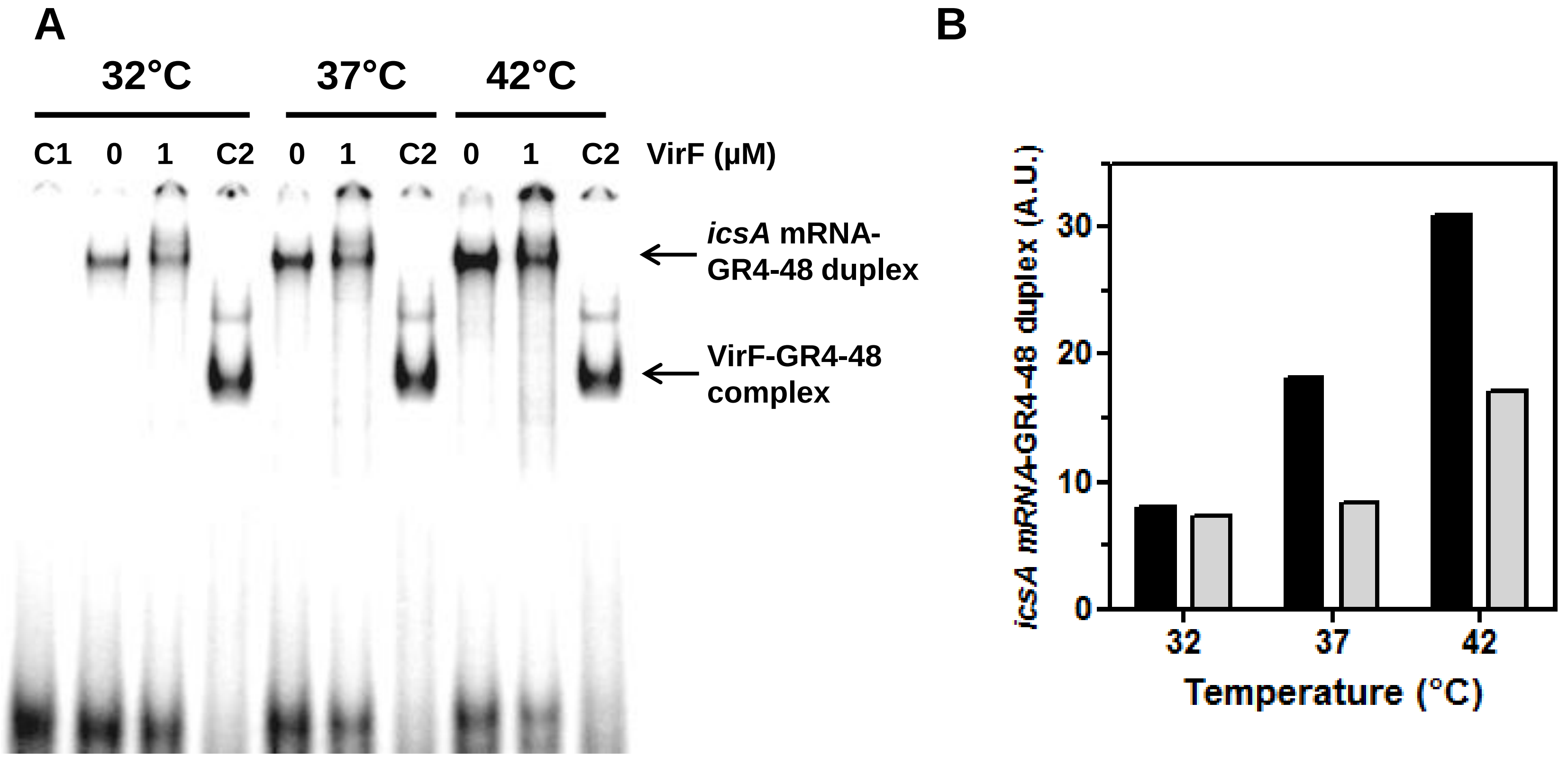

Figure S4. VirF affects the interaction of the GH1 domain of RnaG with *icsA* mRNA as a function of temperature. **A**. Pairing Assay (RRPA) was performed at 32, 37 and 42°C as described in *Material and Methods* using VirF (1 µM) and the GR4-48 RNA corresponding to the GH1 domain of RnaG. Lane C1 is a control sample containing the labelled GR4-48 oligo only. Lanes C2 are the control samples, at the indicated temperatures, containing the labelled GR4-48 RNA in presence of VirF (1 µM) but in absence of *icsA370* mRNA. **B.** The radioactivity associated to the ^{32}P-labelled GR4-48-*icsA* mRNA hetero-duplex has been quantified in the absence (back bars) and in the presence (gray bars) of VirF and expressed as Arbitrary Units (A.U.) .

TABLE S1. RNA and DNA oligonucleotides used in this study

Name	Sequence
GR4-48	5'-GUUGAUAAACCCCUGAAAAGAUUCCGUUUUAUCCGGAAUAAAGGG-3'
GR49-80	5'-ACGAUAUAUGCAAAAACAUAUUAAACAAAGCC-3'
M129	5'-AAGUUUUAGUCUUUAUCUAGAGCA-3'
M130	5'-UGCUCUAGAUAAAGACUAGAGCUU-3'
G+110	5'-TTGATAAACCCCTGAAAAGA-3'
G+50	5'-GGTTGAGGCTTTGTTTAATATG-3'
G+1H	5'-CCCAAGCTTGTTGCATTGATATATAACAC-3'
G-100	5'-GAAAGAACTGAAAAGTTGCGG-3'
G+187	5'-CCTGTGAACATTGGGTCAT-3'
VB212	5'-ATCGCGTACATTCGTTTTTTGG-3'
T7VB	5'-CGAATTTAATACGACTCACTATACACTGCATTTAACTTTTGTCAAT-3'
H238	5'-CGGAATTCCCAACAAACCACCCCAATA
H239	5'-TATTAAATTGTCTGGATCCGGACAATAAAA
